# Mapping the availability of climate and health education in European schools of public health: a baseline assessment for indicator development

**DOI:** 10.3389/ijph.2026.1609473

**Published:** 2026-06-26

**Authors:** Eric Phillip Twomey, Fadilah Fitri Arsy, Robert Otok, Laurent Chambaud

**Affiliations:** 1 Department of Epidemiology and Public Health, Swiss Tropical and Public Health Institute, Basel, Switzerland; 2 ASPHER - The Association of Schools of Public Health in the European Region, Brussels, Belgium; 3 Faculty of Science, University of Basel, Basel, Switzerland

**Keywords:** climate change, education, European Region, indicator, public health

## Abstract

**Objectives:**

Climate and health education is increasingly recognised as essential for preparing a climate-competent public health workforce, yet reliable European baseline data remain limited. This cross-sectional study assessed the availability of climate and health education in degree-granting Schools of Public Health across the World Health Organization European Region.

**Methods:**

A survey of Association of Schools of Public Health in the European Region member institutions was combined with a large-language-model-assisted web scan and manual verification of institutional websites, curricula, syllabi, and programme descriptions.

**Results:**

Overall, 268 Schools of Public Health were identified across 53 countries, including 131 ASPHER members and 137 non-members. Of these, 111 institutions (41.4%) offered at least one verified climate and health education programme or course in 2024/2025. Availability was similar among ASPHER members (41%) and non-members (43%). Most identified programmes were offered at master’s level, while vocational, bachelor, doctoral, and continuing professional development opportunities were less frequent.

**Conclusion:**

Climate and health education is present across much of Europe but remains uneven and concentrated at postgraduate level. The findings provide a verifiable baseline for future monitoring and policy action.

## Introduction

Climate change, accelerated by human activity, poses the single greatest threat to human health in the 21st century. The World Health Organization (WHO) estimates 250,000 additional deaths annually between 2030 and 2050 due to climate-related causes such as heat stress, undernutrition, malaria, and diarrhoeal disease [1]. In Europe, the 2024 *Lancet Countdown* reported alarming trends: escalating heat-related mortality, longer periods of unsafe outdoor temperatures, and increasing climate-sensitive infectious disease risks [2]. Despite policy efforts such as the European Green Deal and Health National Adaptation Plans [3, 4], the health sector remains under-prepared, only one-third of countries have early-warning systems for heat-related illness [5]. Strengthening climate-resilient health systems therefore depends on a workforce that is climate-competent.

Public-health professionals play a critical role in building sustainable, climate-resilient systems [6–9]. Competencies include understanding climate–health linkages, applying evidence-based mitigation and adaptation strategies, engaging in multisectoral collaboration, and communicating effectively with communities [10–12]. These skills can only be ensured through formal integration of climate and health (C&H) content into public-health curricula.

Over recent years, interest in C&H education has grown rapidly. The European Green Deal and the 2023 Budapest Declaration of the WHO Ministerial Conference on Environment and Health called on Member States to strengthen climate-related learning [13, 14]. The Association of Schools of Public Health in the European Region (ASPHER) and the European Union (EU) Health Policy Platform further urged integration of planetary-health concepts into education and training with ASPHER’s 2024 Core Curriculum defining six essential competencies in Planetary, One, environmental, and climate health [15]. In a first attempt to quantify C&H education availability in Europe, ASPHER reported that 29 of 45 (64%) respondents of a survey on ASPHER-member Schools of Public Health (SPH) in 2020 offered C&H education programmes [8]. More recently, the *Lancet Countdown on health and climate-change 2024* indicator *“2.2.6 – Climate and Health Education and Training”* reported the number of institutions offering relevant programmes has expanded: globally, 70% of public-health degree-granting institutions now report including C&H education [5]. This indicator was based on previous work by Sorensen et al. [9], who, in the 53 countries of the WHO European Region (WHO-ER53), invited 155 degree-awarding public health institutions to provide information on C&H education availability. As institutions use diverse labels, such as *School*, *Institute*, *Faculty*, *Department*, or *Centre* of Public Health, the authors used a pragmatic definition of their denominator by considering all institutions awarding degrees in public health as eligible but did not specify how institutions unknown to the authors were identified. Of the 155 institutions invited, 66 institutions responded, representing 27 (∼51%) of the 53 WHO-ER53 countries. Among the responding subsample, 53 institutions reported offering C&H education, yielding an estimated C&H education availability of 80%. This figure is based on responding institutions rather than the full invitation denominator, adding to several methodological limitations: First, as mentioned, its denominator is uncertain, based on a subsample and thus likely unstable. To our knowledge, to this day, there is no comprehensive and verified count of European institutions awarding degrees in public health. Therefore, the proportion of institutions offering C&H education cannot be reliably tracked over time. As of 2024, ASPHER’s member SPH alone made up 131 institutions across the WHO-ER53, almost twice the number used in Sorensen et al.’s indicator, suggesting a possible under-coverage and non-representativeness. Second, Sorensen et al.’s survey relied on voluntary participation, introducing a possible selection and network bias. Institutions already active in C&H education, and those better connected to the English-speaking Global Consortium on Climate and Health Education (GCCHE), can be assumed to be more likely to respond. Third, the data were not independently validated, as no cross-checking of institutional curricula or websites was done, making them vulnerable to a social-desirability bias. Finally, no common definition of what C&H education constitutes was given. Within Sorensen’s survey instrument, the identification was left to the responding individual, possibly introducing classification bias. C&H education offerings range from single lectures or seminars to full modules or dedicated degrees. Many are embedded within broader frameworks such as Planetary Health or One Health, which address C&H intersections to varying [16, 17]. Environmental Health, as a standalone framework, has traditionally focused on physical, chemical and biological pollution as well as sanitation [18] and therefore should only be considered to provide education on C&H if this is stated as such explicitly. The reported 80% C&H education availability in the WHO-ER53 may overestimate true availability.

To address these limitations of previous attempts to establish a reliable and stable indicator on C&H education across Europe that is useful for policy monitoring and future scientific evaluation, both the denominator and numerator must [1] be based on a common and consistent definition [2], include previously unidentified degree-awarding institutions and C&H programmes and [3] be externally verified. To establish such an indicator, the present study combined results from a survey of ASPHER member SPH with an independent web-scan of institutional websites thus generating a comprehensive and verifiable overview of C&H education in SPH across Europe. Specifically, the study aimed to:

1. Identify all degree-granting public-health institutions across three administrative definitions of Europe (WHO-ER53; EEA-32; EU-27).
2. Determine which SPH provide C&H education in 2024/2025 based on survey responses and publicly verifiable programme information; and
3. Examine how this provision varied by ASPHER membership status, educational level, and regional classification.

The findings of this study are intended to support future longitudinal assessments and to inform educational policy, accreditation, and workforce development in the context of climate-resilient public health systems.

## Methods

### Study design

In order to establish an improved indicator on climate and health (C&H) education by Schools of Public Health (SPH) in the 53 countries of the WHO European Region (WHO-ER53), this cross-sectional study first quantified a stable denominator adopting Sorensen’s pragmatic definition of a SPH as *“institutions offering degree-level education in public health, irrespective of nomenclature”*. Then it established a verified numerator on C&H programmes by quantifying all structured public-health programmes containing disclosed climate- and health-related content were considered part of C&H education.

**FIGURE 1:**
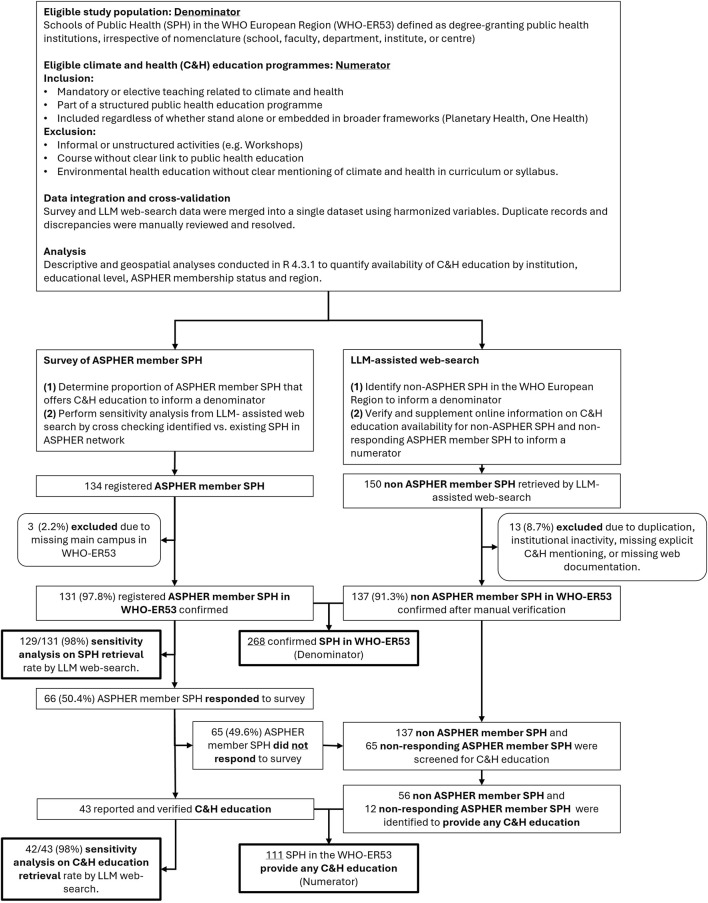
Overview of the data collection, verification, and integration process used to identify climate and health education in Schools of Public Health (SPH) in the World Health Organization European Region (WHO-ER53), 2024/2025. The study entity comprised degree-granting SPH in the WHO-ER53, irrespective of institutional nomenclature (e.g., school, faculty, department, institute, or centre). Data were collected through two complementary pathways: (1) a survey of Association of Schools of Public Health in the European Region (ASPHER) member SPH and (2) a large language model (LLM)-assisted website-scan. The survey was used to identify climate and health education among ASPHER member institutions, while the website review was used to identify non-member SPH and to verify and Supplementary Material for responding and non-responding member institutions. Institutions were eligible if they were located in the WHO-ER53 and awarded a degree in public health. Climate and health (C&H) education was counted when mandatory or elective teaching related to climate and health was part of a structured public health education programme, either as a stand-alone offer or embedded within broader frameworks such as Planetary Health or One Health. Informal or unstructured activities, courses without a clear link to public health education, and environmental health teaching without explicit climate and health content were not counted. Survey and website data were then merged, manually cross-validated, and analysed to assess climate and health education availability by institution, educational level, ASPHER membership status, and region.

Data from [1] a survey on C&H education provision among ASPHER members (Supplementary Table S4) were combined with [2] a complementary Large-Language-Model (LLM)-assisted web scan (Figure 1). For the identification of C&H education, both mandatory and elective courses were considered, whether standalone or integrated within broader curricula. Education levels included vocational, bachelor, master, doctoral, and continuing professional development (CPD) programmes. Informal or non-structured activities such as workshops were excluded. The survey also assessed alignment with the *ASPHER Core Curriculum for Public Health on Planetary, Environmental, and Climate Health Competencies* [15]. ASPHER membership status was retained as an analytic variable. This was done as previous European studies of C&H education were based primarily on ASPHER-linked or network-based samples, and membership in ASPHER may reflect greater institutional connectedness to regional curriculum development, competency frameworks, and inter-school exchange. Stratifying institutions by ASPHER membership therefore allowed a descriptive comparison between network-affiliated and non-affiliated SPH.

#### ASPHER survey design and data collection

As of 2024, ASPHER counted 134 full member institutions, of which 131 ASPHER member SPH with their main campus based within the WHO-ER53 were invited to report programmes integrating elements of *climate and health*, *Planetary Health*, or *One Health*. Data collection took place between April and June 2025, using standardised email invitations via the ASPHER Secretariat. Institutions received reminders and, where necessary, short clarification meetings were arranged with institutional representatives (Figure 1). The questionnaire, based partially on Sorensen et al. [9], covered four thematic areas: (1) institutional information, (2) availability of C&H education, (3) programme details across education levels, and (4) supporting resources. For each reported programme, the survey collected the title, short description, education level, course type, timing, duration, European Credit Transfer and Accumulation System credits (ECTS) where applicable, degree or certificate awarded, language of instruction, student enrolment, enrolment structure, delivery format, cost, competency coverage, and URL; additional questions assessed planned future programmes, implementation challenges, funding, and availability of institutional expertise. A more detailed survey instrument can be found in Supplementary Table S4. Each reported C&H education activity was manually reviewed and verified.

#### LLM-assisted institutional identification and verification

To verify responses and to complement data from the ASPHER survey with data on non-members and non-responding ASPHER members, an LLM-assisted retrieval step was used to generate preliminary country-level lists of potentially eligible institutions and to identify publicly available programme documentation. Searches were conducted between April and June 2025 using GPT-4o (OpenAI; paid subscription version with web-browsing/lookup enabled). The model was used only as a screening and retrieval aid, not for final classification or data extraction. For each WHO-ER53 country, structured prompts requested institutions offering degree-awarding public health education at vocational, bachelor, master, doctoral, or continuing professional development (CPD) level, excluding already known ASPHER members where applicable. Outputs were recorded in tabular form, including country, institution name, programme name, education level, and source links (Figure 1).

All LLM outputs were manually verified by the authors using official institutional websites and publicly available programme pages, curricula, syllabi, or course descriptions. Institutions were retained only when degree-awarding public health education could be confirmed from official sources. C&H education was recorded when explicit C&H, Planetary Health or One Health content was found in relation to a public health programme or an elective course offered by a public health or health-related school, department, or faculty. Offerings of environmental health education needed explicit reference to C&H to be included because the framework’s coverage of C&H topics is uncertain [18]. Duplicates, unsupported links, inconsistent entries, and institutions without verifiable public health degree provision were corrected or excluded. Websites in languages other than English were assessed using browser- and LLM-based translation tools, and ambiguous cases were resolved by repeated manual checking of the original institutional source. The full prompt protocol and verification workflow are provided in Supplementary Material 1. Verified information was extracted according to variables matching the ASPHER Survey 2025 to ensure consistency across datasets. Duplicate and missing data were manually reviewed and corrected during cleaning.

### Data analysis

All analyses were conducted in R 4.3.1 [19]. Survey and web-scan-derived data were cross-validated to resolve discrepancies and enrich institutional profiles. Descriptive statistics quantified the proportion of SPH offering C&H education in 2024/2025, disaggregated by education level. Geospatial analysis illustrated regional disparities and visualised the density of programmes by country.

## Results

### Identified schools of public health–denominator

Through the Large-Language-Model (LLM)-assisted web retrieval, across the World Health Organization European Region (WHO-ER53), a total of 150 Schools of Public Health (SPH) outside of the Association of Schools of Public Health (ASPHER) network were identified. After manual verification, 13 (8.7%) were excluded due to duplication, institutional inactivity, or missing web documentation; 137 SPH (91.3%) were confirmed manually. Of the 134 listed ASPHER member SPH, 131 SPH had main campuses based in the WHO-ER53 and were invited to participate. Through the combination of ASPHER members’ list and the LLM-assisted web-scan representing all 53 countries of the WHO European Region 268 SPH were identified as the denominator for all subsequent analyses within WHO-ER53: 131 (49%) being ASPHER member institutions and 137 (51%) non-member institutions (Table 1). Most SPH were observed in the United Kingdom (n = 33), Germany (n = 15), Turkey (n = 14), Poland (n = 12) and Italy (n = 11) hosting the largest numbers. In contrast, Southeastern and Eastern European countries, such as Albania (n = 1), Montenegro (n = 1), Tajikistan (n = 1) and Armenia (n = 3), had comparatively few SPH offering degree-awarding public-health programmes.

**TABLE 1:** Climate and health education availability at Schools of Public Health in the World Health Organization European Region by member status at the Association of Schools of Public Health in the European Region, 2024/2025.

Climate and health education availability in Schools of Public Health in the WHO European Region						
Counts and percentages by country						
Country	Total SPH	ASPHER SPH n (%)	Non-ASPHER SPH n (%)	Total C&H Ed	ASPHER SPH C&H Ed n (%)	Non-ASPHER C&H Ed SPH n (%)
Total WHO-ER53	268	131 (48.9%)	137 (51.1%)	111 (41.4%)	55 (42.0%)	56 (40.9%)
United Kingdom	33	15 (45.5%)	18 (54.5%)	14 (42.4%)	8 (53.3%)	6 (33.3%)
Germany	15	13 (86.7%)	2 (13.3%)	8 (53.3%)	7 (53.8%)	1 (50.0%)
Turkey	14	1 (7.1%)	13 (92.9%)	4 (28.6%)	0 (0.0%)	4 (30.8%)
Poland	12	8 (66.7%)	4 (33.3%)	4 (33.3%)	2 (25.0%)	2 (50.0%)
Italy	11	7 (63.6%)	4 (36.4%)	5 (45.5%)	4 (57.1%)	1 (25.0%)
Spain	9	5 (55.6%)	4 (44.4%)	4 (44.4%)	1 (20.0%)	3 (75.0%)
Ireland	8	3 (37.5%)	5 (62.5%)	4 (50.0%)	2 (66.7%)	2 (40.0%)
Portugal	8	6 (75.0%)	2 (25.0%)	3 (37.5%)	1 (16.7%)	2 (100.0%)
Sweden	8	4 (50.0%)	4 (50.0%)	5 (62.5%)	2 (50.0%)	3 (75.0%)
Austria	7	4 (57.1%)	3 (42.9%)	3 (42.9%)	2 (50.0%)	1 (33.3%)
France	7	3 (42.9%)	4 (57.1%)	3 (42.9%)	1 (33.3%)	2 (50.0%)
Georgia	7	3 (42.9%)	4 (57.1%)	4 (57.1%)	2 (66.7%)	2 (50.0%)
Netherlands	7	4 (57.1%)	3 (42.9%)	4 (57.1%)	3 (75.0%)	1 (33.3%)
Bulgaria	6	4 (66.7%)	2 (33.3%)	1 (16.7%)	1 (25.0%)	0 (0.0%)
Kazakhstan	6	5 (83.3%)	1 (16.7%)	1 (16.7%)	0 (0.0%)	1 (100.0%)
Belgium	5	1 (20.0%)	4 (80.0%)	3 (60.0%)	0 (0.0%)	3 (75.0%)
Czechia	5	3 (60.0%)	2 (40.0%)	1 (20.0%)	0 (0.0%)	1 (50.0%)
Denmark	5	2 (40.0%)	3 (60.0%)	2 (40.0%)	1 (50.0%)	1 (33.3%)
Israel	5	5 (100.0%)	0 (0.0%)	2 (40.0%)	2 (40.0%)	0 (0.0%)
Lithuania	5	3 (60.0%)	2 (40.0%)	1 (20.0%)	1 (33.3%)	0 (0.0%)
Norway	5	1 (20.0%)	4 (80.0%)	2 (40.0%)	1 (100.0%)	1 (25.0%)
Ukraine	5	1 (20.0%)	4 (80.0%)	3 (60.0%)	0 (0.0%)	3 (75.0%)
Cyprus	4	3 (75.0%)	1 (25.0%)	3 (75.0%)	3 (100.0%)	0 (0.0%)
Estonia	4	1 (25.0%)	3 (75.0%)	2 (50.0%)	1 (100.0%)	1 (33.3%)
Finland	4	2 (50.0%)	2 (50.0%)	3 (75.0%)	2 (100.0%)	1 (50.0%)
Greece	4	2 (50.0%)	2 (50.0%)	3 (75.0%)	1 (50.0%)	2 (100.0%)
Kyrgyzstan	4	0 (0.0%)	4 (100.0%)	1 (25.0%)	0 (0.0%)	1 (25.0%)
Malta	4	1 (25.0%)	3 (75.0%)	1 (25.0%)	1 (100.0%)	0 (0.0%)
Romania	4	3 (75.0%)	1 (25.0%)	1 (25.0%)	0 (0.0%)	1 (100.0%)
Russia	4	0 (0.0%)	4 (100.0%)	0 (0.0%)	0 (0.0%)	0 (0.0%)
Serbia	4	2 (50.0%)	2 (50.0%)	2 (50.0%)	1 (50.0%)	1 (50.0%)
Slovakia	4	2 (50.0%)	2 (50.0%)	2 (50.0%)	0 (0.0%)	2 (100.0%)
Slovenia	4	1 (25.0%)	3 (75.0%)	1 (25.0%)	0 (0.0%)	1 (33.3%)
Armenia	3	2 (66.7%)	1 (33.3%)	1 (33.3%)	1 (50.0%)	0 (0.0%)
Hungary	3	1 (33.3%)	2 (66.7%)	2 (66.7%)	1 (100.0%)	1 (50.0%)
Switzerland	3	1 (33.3%)	2 (66.7%)	2 (66.7%)	1 (100.0%)	1 (50.0%)
Uzbekistan	3	0 (0.0%)	3 (100.0%)	1 (33.3%)	0 (0.0%)	1 (33.3%)
Belarus	2	0 (0.0%)	2 (100.0%)	0 (0.0%)	0 (0.0%)	0 (0.0%)
Bosnia and Herzegovina	2	0 (0.0%)	2 (100.0%)	1 (50.0%)	0 (0.0%)	1 (50.0%)
Croatia	2	1 (50.0%)	1 (50.0%)	1 (50.0%)	0 (0.0%)	1 (100.0%)
Iceland	2	1 (50.0%)	1 (50.0%)	1 (50.0%)	1 (100.0%)	0 (0.0%)
Latvia	2	1 (50.0%)	1 (50.0%)	0 (0.0%)	0 (0.0%)	0 (0.0%)
Albania	1	1 (100.0%)	0 (0.0%)	0 (0.0%)	0 (0.0%)	0 (0.0%)
Andorra	1	0 (0.0%)	1 (100.0%)	1 (100.0%)	0 (0.0%)	1 (100.0%)
Kosovo	1	1 (100.0%)	0 (0.0%)	0 (0.0%)	0 (0.0%)	0 (0.0%)
Luxembourg	1	0 (0.0%)	1 (100.0%)	0 (0.0%)	0 (0.0%)	0 (0.0%)
Moldova	1	1 (100.0%)	0 (0.0%)	1 (100.0%)	1 (100.0%)	0 (0.0%)
Montenegro	1	1 (100.0%)	0 (0.0%)	0 (0.0%)	0 (0.0%)	0 (0.0%)
North Macedonia	1	1 (100.0%)	0 (0.0%)	0 (0.0%)	0 (0.0%)	0 (0.0%)
Palestine	1	1 (100.0%)	0 (0.0%)	0 (0.0%)	0 (0.0%)	0 (0.0%)
Tajikistan	1	0 (0.0%)	1 (100.0%)	0 (0.0%)	0 (0.0%)	0 (0.0%)

### Availability of climate and health education - numerator

From the 131 invited ASPHER member SPH, 66 (50.4%) institutions responded to the survey. Among these, 43 reported offering C&H education and 23 reported none. The remaining 65 (40.6%) non-responding ASPHER member institutions were included in the LLM-assisted scan. Among these, only 12 (18.5%) were identified with available C&H education. Out of the combined 268 ASPHER and non-ASPHER member SPH, 111 (41%) were found to offer at least one C&H education programme or related course (Table 1). Among ASPHER members, 55 out of 131 institutions (42%) reported or were identified as providing C&H education, while in non-member institutions 56 out of 137 (41%) incorporated C&H content, either stand-alone or within broader frameworks such as Planetary Health, One Health, or environmental health.

**FIGURE 2:**
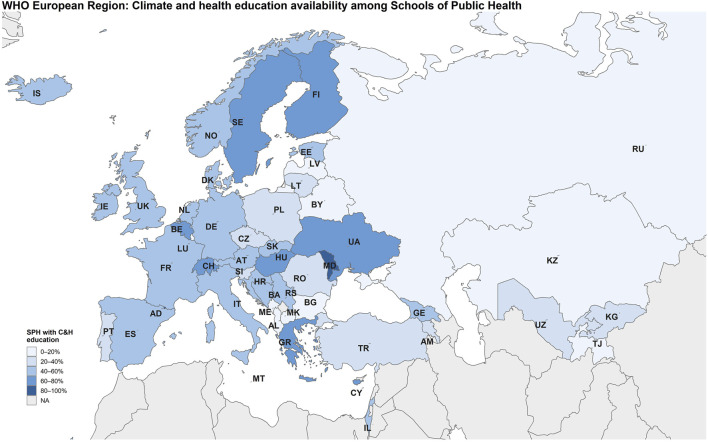
Country-level availability of climate and health (C&H) education among Schools of Public Health (SPH) in the World Health Organization European Region (WHO-ER53), 2024/2025. The map shows the proportion of identified SPH in each country offering at least one C&H education programme or course in 2024/2025. Shading represents the share of SPH with any C&H education offer by country, grouped into five percentage categories from 0% to 100%.

**FIGURE 3:**
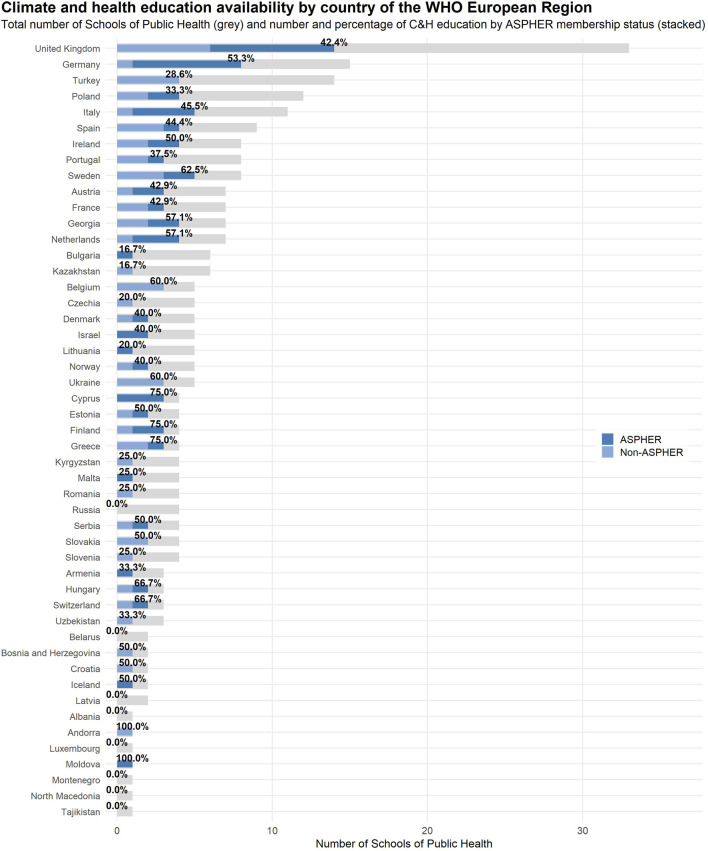
Climate and health education availability by country in the World Health Organization European Region (WHO-ER53), 2024/2025. The figure shows, for each country in the WHO-ER53, the total number of Schools of Public Health (SPH) as grey horizontal bars. Overlaid stacked blue bars show the number of SPH offering at least one climate and health (C&H) education programme, stratified by membership status in the Association of Schools of Public Health in the European Region (ASPHER). Percentage labels indicate the proportion of SPH in each country that offer at least one C&H education programme. Countries are ordered by the total number of SPH.

Within WHO-ER53, C&H education was offered in 45 out of 53 countries (81%); only in eight countries, namely Russia, Belarus, Latvia, Albania, Luxembourg, Montenegro, North Macedonia and Tajikistan, no C&H education was reported or found (Figure 2). In absolute terms, most C&H education programmes were offered in the United Kingdom (n = 14), Germany (n = 8), Italy and Sweden (both 5). In relation to the number of SPH, highest relative coverage of C&H education was offered in Andorra and Moldova (both 100%, 1/1), followed by Greece, Finland and Cyprus (all 75%, ¾) and Hungary and Switzerland (67%, 2/3) (Figure 3).

### Educational level of C&H programmes

Across the 111 institutions offering distinct C&H education programmes, 116 distinct programmes were identified (Supplementary Table S1). Most were taught at master’s level (n = 81; 69.8%), followed by bachelor’s level (n = 14; 13.8%), continuing professional development (CPD)/short courses (n = 11; 9.5%) and doctoral level (n = 9; 7.8%). Only one vocational/technical training was found.

**FIGURE 4:**
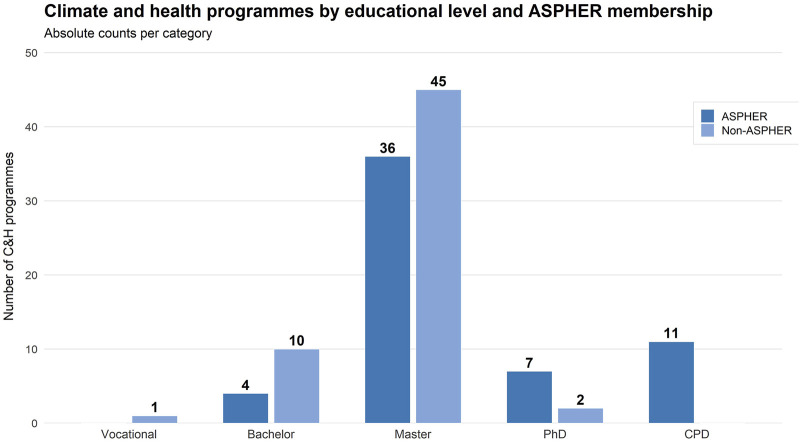
Climate and health programmes by educational level and ASPHER membership, 2024/2025. The figure shows the absolute number of climate and health programmes by educational level and membership in the Association of Schools of Public Health in the European Region (ASPHER). In total, 116 programmes were counted across Schools of Public Health (SPH). Most programmes were offered at master’s level, with 81 programmes (69.8%), followed by bachelor’s level with 14 programmes (12.1%), continuing professional development (CPD) with 11 programmes (9.5%), doctoral (PhD) level with 9 programmes (7.8%), and vocational level with 1 programme (0.9%). Overall, 106 SPH (95.5%) offered only one counted programme, whereas 5 SPH (4.5%) offered more than one counted programme. Bars are stratified by ASPHER and non-ASPHER membership.

C&H content showed the highest availability in postgraduate curricula with 101 out of 116 (87.1%), often as elective or thematic modules within Master of Public Health (MPH) degrees, while undergraduate and vocational courses tended to include brief sessions on climate-related determinants of health. When comparing SPH by ASPHER membership status, slightly more postgraduate (n = 54) offerings of C&H programmes were identified among ASPHER members compared to non-members (n = 47). On vocational or bachelor levels, however, non-members showed more offerings (n = 11) than ASPHER members (n = 4) (Figure 4).

### Regional disparities

In absolute terms, Western-, Northern and Southern Europe show most numbers of SPH (Figure 3). Looking at relative C&H education availability, however, contrary to prior expectations, there is no clear regional gradient or disparity to be detected. Data were also descriptively analysed by varying definition of European Regions, i.e. EU-27, EEA-32, WHO-ER53 (Supplementary Tables S2, S3). Availability of C&H education programmes did not differ substantiallybetween European administrative definitions, ranging from 41% to 47% within non-members and 42%–43% within ASPHER members (Supplementary Figure S1).

## Discussion

This study presents the first comprehensive, region-wide dataset on climate and health (C&H) education in Schools of Public Health (SPH) across the World Health Organization European Region (WHO-ER53), European Union (EU-27) and European Environment Agency member countries (EEA-32). Using a combined survey and Large-Language-Model (LLM)-assisted web-scan approach, 268 degree-granting SPH were identified, and 111 (41%) were found to offer one or more C&H-related programmes in 2024/2025. This coverage is approximately half of previous estimates reported by Sorensen et al. [9] and used in the *Lancet Countdown Europe 2024 indicator 2.2.6* [5], which had suggested that up to 80% of European institutions provided C&H education. The present findings provide a more transparent and externally verifiable baseline, while remaining subject to residual retrieval bias, incomplete public web documentation, language-related limitations, and variation in institutional transparency, highlighting some gaps and disparities on educational and geographical levels. In absolute terms, programmes are most frequently found in Western, Northern and Southern Europe, but absolute coverage remains limited in Eastern and Southeastern Europe. In relative terms, however, the proportion of SPH offering C&H education is similar across subregions once institutional density is accounted for. This pattern is consistent with structural differences in SPH distribution, but the study cannot assess institutional commitment. Smaller countries such as Moldova, Greece, and Finland show high proportional integration; the reasons for this pattern require further investigation. In this study, Western and Northern European countries host the largest absolute number of SPH and the highest counts of C&H programmes in absolute terms, however, these regions are generally less exposed to climate risk than parts of Southern and Eastern Europe, which face higher projected temperature rises and vulnerability indices (European Commission INFORM Index, 2024). In this dataset, some regions with high climate vulnerability also had lower absolute numbers of identified SPH and documented C&H education programmes. This descriptive mismatch between climate risk and documented educational coverage is policy-relevant, but it cannot establish causal relationships or institutional capacity differences. It should be interpreted as a hypothesis-generating observation warranting further investigation using more detailed institutional, workforce, and exposure data.

C&H education in Europe is still concentrated at postgraduate level, predominantly within Master of Public Health (MPH) programmes, while vocational, undergraduate, and doctoral training opportunities remain rare. This imbalance limits early-stage exposure to climate-related health concepts and may delay skill acquisition that is critical for health-system resilience. The scarcity of vocational and technical programmes represents a missed opportunity, as graduates from these pathways often play frontline roles in implementing adaptation and preparedness measures [20]. Furthermore, including availability and quality of C&H education would be a valuable addition to the 2024 newly established model for ranking schools of public health [21].

In comparison with previous studies aiming to estimate C&H education availability across Europe, the lower availability observed here can be explained by methodological differences with earlier studies. Both Orhan et al. [8] and Sorensen et al. [9] relied on a voluntary survey with potential selection, response, network, social-desirability and language biases toward English-speaking and internationally connected institutions, likely inflating estimates. This notion of a response bias is corroborated by the finding of differential C&H education availability between ASPHER survey respondents and non-respondents. Further, Sorensen and colleagues based their estimates on 66 out of 155 responding institutions from only 27 out of 53 countries of the WHO-ER53. In contrast, the present study used an independently assessed denominator, based on verifiable public information encompassing all known European SPH, both ASPHER member and non-member institutions, and cross-validated survey data with publicly accessible programme descriptions. The resulting figure of 41% should therefore be interpreted as a conservative, externally verifiable estimate of documented C&H education availability, rather than as a definitive measure of all existing provision.

Consistent with previous work [8, 22], most identified programmes integrate climate content under broader frameworks such as Planetary Health, One Health, or environmental health. This confirms that while C&H themes are increasingly acknowledged, they are rarely formalised as standalone curricula. Programmes focusing exclusively on C&H remain limited, suggesting the field is still in an early institutionalisation phase within European higher education.

Although our study did not directly assess barriers or facilitators to integrating C&H education, evidence from previous research provides plausible explanations for the comparatively low availability. Prior studies have highlighted constraints such as limited funding, insufficient faculty expertise, lack of curricular mandates, and competing institutional priorities as major obstacles to integration [8, 23]. Additional structural factors, such as linguistic and political diversity across Europe, absence of coordinated national guidance, and weak inter-institutional collaboration, may further hinder progress. Similar barriers have been reported in other regions: in the United States, administrative support, funding availability, and faculty capacity were identified as main determinants of successful curriculum implementation [6, 23]. Moreover, European climate-adaptation strategies rarely include concrete measures to strengthen the health workforce, focusing instead on infrastructure and biomedical interventions [24]. However, while LLM-assistance helped overcome language barriers during the data collection, a lack of regionally specific literature, especially on Central Asian and Eastern European countries must be acknowledged. Barriers to and facilitators of C&H education implementation might vary in these countries. Taken together, these explanations remain speculative in the context of our study and warrant systematic investigation at the European level. Understanding such barriers will be essential for policymakers seeking to design effective strategies to expand and institutionalise C&H education across SPH.

Comprehensive C&H education is essential for developing climate-competent health workforces, one of the pillars of WHO’s operational framework for building climate-resilient and low-carbon health systems [14]. Public-health professionals must be equipped not only with scientific understanding but also with the ability to advocate, communicate, and design multisectoral responses to climate threats [11, 15]. The current limited availability of such education in Europe thus signals an ongoing mismatch between European policy ambitions and workforce preparation.

Several limitations must be acknowledged. First, the study is cross-sectional, thus unable to support causal inferences or to depict temporal trends. Future iterations of the indicator will enable longitudinal monitoring to assess policy impact and programme growth and may help explore factors associated with C&H education availability. Second, although the definition of SPH followed an inclusive approach, institutional structures vary considerably across countries, ranging from multi-faculty universities to national umbrella schools (e.g. the Swiss School of Public Health). This variability may affect comparability and introduce classification uncertainty. Third, the LLM-assisted web-scan component depended on publicly available web data, which may be outdated or incomplete. The use of LLM assistance improved coverage but also introduced a potential retrieval bias, which was addressed by a sensitivity validation showing a 98% retrieval rate. However, despite all efforts made to identify all SPH and available C&H programmes, it must be emphasised that some SPH or programmes may have gone undetected by our methodology. Our approach warrants continued methodological evaluation as LLMs are inherently probabilistic; and undergo continuous modification; exact reproducibility cannot be guaranteed as in classical systematic searches. Further, the study did not evaluate course quality and extensiveness (ECTS), faculty qualifications, alignment with the ASPHER Core Curriculum, student numbers and participation, language of instruction, institutional engagement, economic capacity or political commitment. Inferences based on these elements are warranted in future iterations of this indicator. Finally, it excluded other health-education domains such as medicine, nursing, and pharmacy, where climate-related training is also highly relevant. Expanding future surveys to include these dimensions and these disciplines will be essential for a full picture of Europe’s climate-health workforce capacity.

Despite these limitations, this study makes two major contributions by first, providing a stable and defensible denominator of degree-awarding public-health institutions across Europe, enabling future calculation of standardised indicators. Second, it establishes a transparent, reproducible methodology combining LLM-assisted searches with manual verification, adaptable for replication on other curricula and in other WHO regions. Collectively, this monitoring tool and baseline assessment could detect future trends and help inform policy alignment with the European Green Deal, the WHO Operational Framework for Climate-Resilient Health Systems, and the Lancet Countdown Europe indicator set.

The findings underscore that Europe’s progress toward a climate-smart public health workforce, while substantial, is likely smaller than previously reported and remains uneven. Achieving comprehensive integration of climate and health into public-health education and training might be supported through:

1. Mandate climate-health competencies through national accreditation frameworks and accreditation bodies.
2. Funding and faculty-training programmes to build teaching capacity.
3. Regional collaboration through ASPHER and WHO Europe to exchange materials, share best practices, and harmonise terminology.
4. Systematic, continuous monitoring using the ASPHER Repository to track progress annually and guide evidence-based policy.
5. Expansion of this methodology to other, similar frameworks as Planetary Health or One Health and schools of medicine, nursing, pharmacy and paramedical sciences.

Future research should evaluate not only the presence but also the quality and outcomes of C&H education as well as institutional conditions, linking competencies to measurable improvements in workforce preparedness and, ultimately, population resilience to climate change.

## Data Availability

Aggregated data on C&H education availability are publicly accessible through the ASPHER Repository on Climate and Health Education (https://www.aspher.org). De-identified Supplementary Data on supporting resources may be obtained from the corresponding author upon reasonable request.
