# Supplementary material for: Mapping the availability of climate and health education in European schools of public health: a baseline assessment for indicator development
Source: Int J Public Health. 2026 Jun 26;71:1609473. doi: 10.3389/ijph.2026.1609473 (PMC13349935; doi:10.3389/ijph.2026.1609473)
Supplement: Supplementary file 1 [file Supplementaryfile1.docx]

## **Supplementary Material**

*Supplementary Table 1 Climate and health education by levels and membership status at the Association of Schools of Public Health in the European Region in the World Health Organization European Region*

| Category | Total n | Vocational n(%) | Bachelor n(%) | Master n(%) | Doctoral n(%) | CPD n(%) |
| --- | --- | --- | --- | --- | --- | --- |
| ASPHER SPH | 58 | 0 (0.0%) | 4 (6.9%) | 36 (62.1%) | 7 (12.1%) | 11 (19.0%) |
| non-ASPHER SPH | 58 | 1 (1.7%) | 10 (17.2%) | 45 (77.6%) | 2 (3.4%) | 0 (0.0%) |
| Total | 116 | 1 (0.9%) | 14 (12.1) | 81 (69.8%) | 11 (9.5%) | 11 (9.5%) |

*Supplementary Table 2 Climate and health education availability in the 27 member countries of the European Union (EU-27) by membership status at the Association of Schools of Public Health in the European Region*

| **Climate and health education in Schools of Public Health in the European Union (EU-27)** | | | | | | |
| --- | --- | --- | --- | --- | --- | --- |
| Counts and percentages by country (TOTAL at top; n (%) format) | | | | | | |
| **Country** | **Total SPH** | **ASPHER SPH n (%)** | **Non-ASPHER SPH n (%)** | **Total C&H Ed** | **ASPHER SPH C&H Ed n (%)** | **Non-ASPHER C&H Ed SPH n (%)** |
| **Total EU-27** | **158** | **88 (55.7%)** | **70 (44.3%)** | **70 (44.3%)** | **37 (42.0%)** | **33 (47.1%)** |
| Germany | 15 | 13 (86.7%) | 2 (13.3%) | 8 (53.3%) | 7 (53.8%) | 1 (50.0%) |
| Poland | 12 | 8 (66.7%) | 4 (33.3%) | 4 (33.3%) | 2 (25.0%) | 2 (50.0%) |
| Italy | 11 | 7 (63.6%) | 4 (36.4%) | 5 (45.5%) | 4 (57.1%) | 1 (25.0%) |
| Spain | 9 | 5 (55.6%) | 4 (44.4%) | 4 (44.4%) | 1 (20.0%) | 3 (75.0%) |
| Ireland | 8 | 3 (37.5%) | 5 (62.5%) | 4 (50.0%) | 2 (66.7%) | 2 (40.0%) |
| Portugal | 8 | 6 (75.0%) | 2 (25.0%) | 3 (37.5%) | 1 (16.7%) | 2 (100.0%) |
| Sweden | 8 | 4 (50.0%) | 4 (50.0%) | 5 (62.5%) | 2 (50.0%) | 3 (75.0%) |
| Austria | 7 | 4 (57.1%) | 3 (42.9%) | 3 (42.9%) | 2 (50.0%) | 1 (33.3%) |
| France | 7 | 3 (42.9%) | 4 (57.1%) | 3 (42.9%) | 1 (33.3%) | 2 (50.0%) |
| Netherlands | 7 | 4 (57.1%) | 3 (42.9%) | 4 (57.1%) | 3 (75.0%) | 1 (33.3%) |
| Bulgaria | 6 | 4 (66.7%) | 2 (33.3%) | 1 (16.7%) | 1 (25.0%) | 0 (0.0%) |
| Belgium | 5 | 1 (20.0%) | 4 (80.0%) | 3 (60.0%) | 0 (0.0%) | 3 (75.0%) |
| Czechia | 5 | 3 (60.0%) | 2 (40.0%) | 1 (20.0%) | 0 (0.0%) | 1 (50.0%) |
| Denmark | 5 | 2 (40.0%) | 3 (60.0%) | 2 (40.0%) | 1 (50.0%) | 1 (33.3%) |
| Lithuania | 5 | 3 (60.0%) | 2 (40.0%) | 1 (20.0%) | 1 (33.3%) | 0 (0.0%) |
| Cyprus | 4 | 3 (75.0%) | 1 (25.0%) | 3 (75.0%) | 3 (100.0%) | 0 (0.0%) |
| Estonia | 4 | 1 (25.0%) | 3 (75.0%) | 2 (50.0%) | 1 (100.0%) | 1 (33.3%) |
| Finland | 4 | 2 (50.0%) | 2 (50.0%) | 3 (75.0%) | 2 (100.0%) | 1 (50.0%) |
| Greece | 4 | 2 (50.0%) | 2 (50.0%) | 3 (75.0%) | 1 (50.0%) | 2 (100.0%) |
| Malta | 4 | 1 (25.0%) | 3 (75.0%) | 1 (25.0%) | 1 (100.0%) | 0 (0.0%) |
| Romania | 4 | 3 (75.0%) | 1 (25.0%) | 1 (25.0%) | 0 (0.0%) | 1 (100.0%) |
| Slovakia | 4 | 2 (50.0%) | 2 (50.0%) | 2 (50.0%) | 0 (0.0%) | 2 (100.0%) |
| Slovenia | 4 | 1 (25.0%) | 3 (75.0%) | 1 (25.0%) | 0 (0.0%) | 1 (33.3%) |
| Hungary | 3 | 1 (33.3%) | 2 (66.7%) | 2 (66.7%) | 1 (100.0%) | 1 (50.0%) |
| Croatia | 2 | 1 (50.0%) | 1 (50.0%) | 1 (50.0%) | 0 (0.0%) | 1 (100.0%) |
| Latvia | 2 | 1 (50.0%) | 1 (50.0%) | 0 (0.0%) | 0 (0.0%) | 0 (0.0%) |
| Luxembourg | 1 | 0 (0.0%) | 1 (100.0%) | 0 (0.0%) | 0 (0.0%) | 0 (0.0%) |

*Supplementary Table 3: Climate and health education availability in the 32 member countries of the European Environment Agency (EEA-32) by membership status at the Association of Schools of Public Health in the European Region*

| **Climate and health education in Schools of Public Health in the 32 member countries of the European Environment Agency** | | | | | | |
| --- | --- | --- | --- | --- | --- | --- |
| Counts and percentages by country (TOTAL at top; n (%) format) | | | | | | |
| **Country** | **Total SPH** | **ASPHER SPH n (%)** | **Non-ASPHER SPH n (%)** | **Total C&H Ed** | **ASPHER SPH C&H Ed n (%)** | **Non-ASPHER C&H Ed SPH n (%)** |
| **Total EEA-32** | **182** | **92 (50.5%)** | **90 (49.5%)** | **79 (43.4%)** | **40 (43.5%)** | **39 (43.3%)** |
| Germany | 15 | 13 (86.7%) | 2 (13.3%) | 8 (53.3%) | 7 (53.8%) | 1 (50.0%) |
| Turkey | 14 | 1 (7.1%) | 13 (92.9%) | 4 (28.6%) | 0 (0.0%) | 4 (30.8%) |
| Poland | 12 | 8 (66.7%) | 4 (33.3%) | 4 (33.3%) | 2 (25.0%) | 2 (50.0%) |
| Italy | 11 | 7 (63.6%) | 4 (36.4%) | 5 (45.5%) | 4 (57.1%) | 1 (25.0%) |
| Spain | 9 | 5 (55.6%) | 4 (44.4%) | 4 (44.4%) | 1 (20.0%) | 3 (75.0%) |
| Ireland | 8 | 3 (37.5%) | 5 (62.5%) | 4 (50.0%) | 2 (66.7%) | 2 (40.0%) |
| Portugal | 8 | 6 (75.0%) | 2 (25.0%) | 3 (37.5%) | 1 (16.7%) | 2 (100.0%) |
| Sweden | 8 | 4 (50.0%) | 4 (50.0%) | 5 (62.5%) | 2 (50.0%) | 3 (75.0%) |
| Austria | 7 | 4 (57.1%) | 3 (42.9%) | 3 (42.9%) | 2 (50.0%) | 1 (33.3%) |
| France | 7 | 3 (42.9%) | 4 (57.1%) | 3 (42.9%) | 1 (33.3%) | 2 (50.0%) |
| Netherlands | 7 | 4 (57.1%) | 3 (42.9%) | 4 (57.1%) | 3 (75.0%) | 1 (33.3%) |
| Bulgaria | 6 | 4 (66.7%) | 2 (33.3%) | 1 (16.7%) | 1 (25.0%) | 0 (0.0%) |
| Belgium | 5 | 1 (20.0%) | 4 (80.0%) | 3 (60.0%) | 0 (0.0%) | 3 (75.0%) |
| Czechia | 5 | 3 (60.0%) | 2 (40.0%) | 1 (20.0%) | 0 (0.0%) | 1 (50.0%) |
| Denmark | 5 | 2 (40.0%) | 3 (60.0%) | 2 (40.0%) | 1 (50.0%) | 1 (33.3%) |
| Lithuania | 5 | 3 (60.0%) | 2 (40.0%) | 1 (20.0%) | 1 (33.3%) | 0 (0.0%) |
| Norway | 5 | 1 (20.0%) | 4 (80.0%) | 2 (40.0%) | 1 (100.0%) | 1 (25.0%) |
| Cyprus | 4 | 3 (75.0%) | 1 (25.0%) | 3 (75.0%) | 3 (100.0%) | 0 (0.0%) |
| Estonia | 4 | 1 (25.0%) | 3 (75.0%) | 2 (50.0%) | 1 (100.0%) | 1 (33.3%) |
| Finland | 4 | 2 (50.0%) | 2 (50.0%) | 3 (75.0%) | 2 (100.0%) | 1 (50.0%) |
| Greece | 4 | 2 (50.0%) | 2 (50.0%) | 3 (75.0%) | 1 (50.0%) | 2 (100.0%) |
| Malta | 4 | 1 (25.0%) | 3 (75.0%) | 1 (25.0%) | 1 (100.0%) | 0 (0.0%) |
| Romania | 4 | 3 (75.0%) | 1 (25.0%) | 1 (25.0%) | 0 (0.0%) | 1 (100.0%) |
| Slovakia | 4 | 2 (50.0%) | 2 (50.0%) | 2 (50.0%) | 0 (0.0%) | 2 (100.0%) |
| Slovenia | 4 | 1 (25.0%) | 3 (75.0%) | 1 (25.0%) | 0 (0.0%) | 1 (33.3%) |
| Hungary | 3 | 1 (33.3%) | 2 (66.7%) | 2 (66.7%) | 1 (100.0%) | 1 (50.0%) |
| Switzerland | 3 | 1 (33.3%) | 2 (66.7%) | 2 (66.7%) | 1 (100.0%) | 1 (50.0%) |
| Croatia | 2 | 1 (50.0%) | 1 (50.0%) | 1 (50.0%) | 0 (0.0%) | 1 (100.0%) |
| Iceland | 2 | 1 (50.0%) | 1 (50.0%) | 1 (50.0%) | 1 (100.0%) | 0 (0.0%) |
| Latvia | 2 | 1 (50.0%) | 1 (50.0%) | 0 (0.0%) | 0 (0.0%) | 0 (0.0%) |
| Luxembourg | 1 | 0 (0.0%) | 1 (100.0%) | 0 (0.0%) | 0 (0.0%) | 0 (0.0%) |

***Supplementary Figure 1. Availability of climate and health education by administrative definition and Association of Schools of Public Health in the European Region (ASPHER) membership status.*** *The figure shows the percentage of Schools of Public Health (SPH) offering at least one climate and health programme, stratified by administrative definition of Europe and by ASPHER membership status. Three definitions are shown: the World Health Organization European Region (WHO-ER53), the European Environment Agency 32 member countries (EEA-32), and the European Union 27 member countries (EU-27). Bar labels report the number of schools with at least one climate and health programme over the total number of schools in that stratum, followed by the corresponding percentage. Counts are based on unique SPH.*


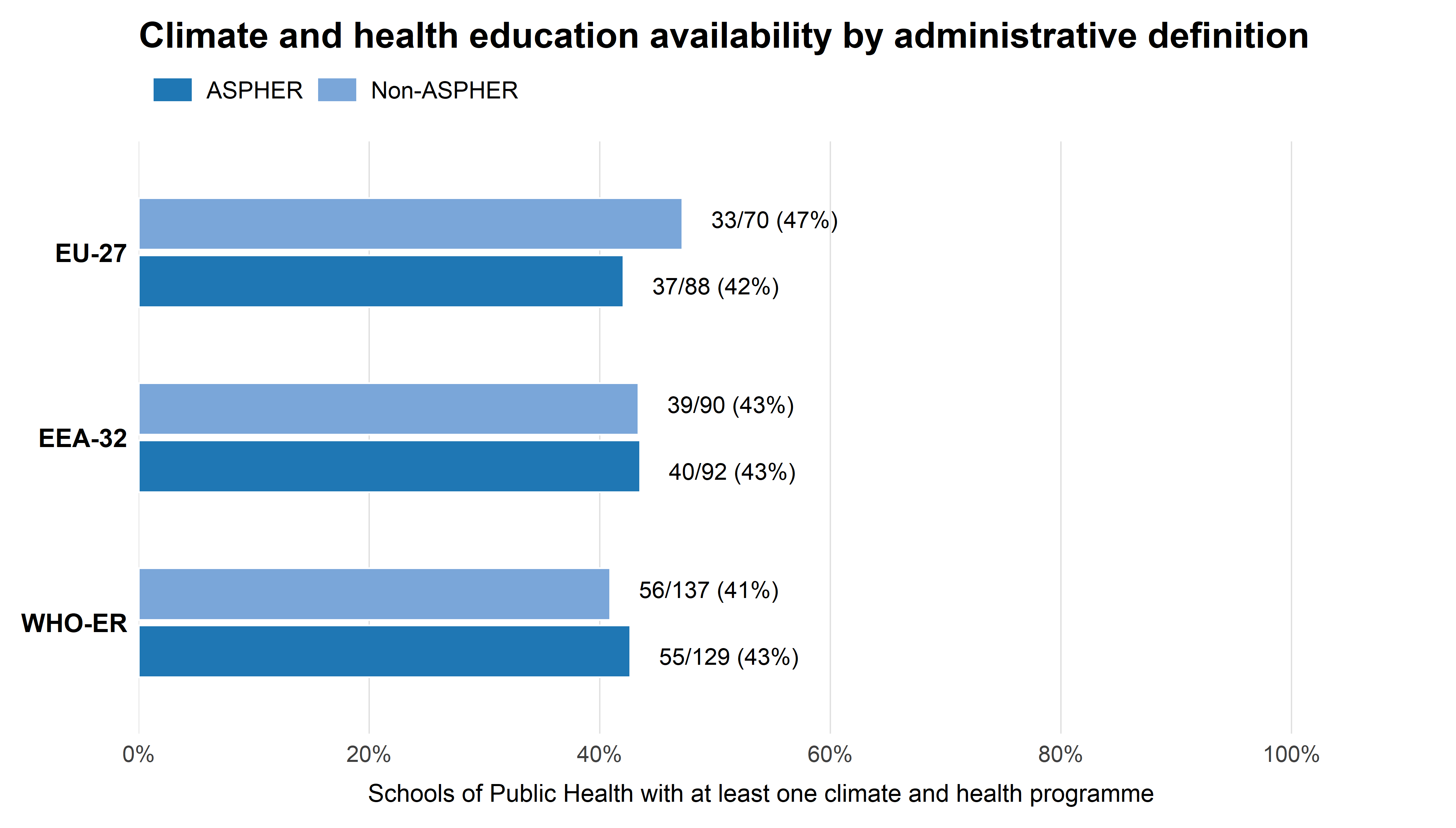


Supplementary Table 4: Association of Schools of Public Health in the European Region Climate and health education survey instrument, simplified table

| ASPHER Climate and health education survey instrument, simplified table | | | | |
| --- | --- | --- | --- | --- |
| **Section** | **Question group** | **Survey item / variable** | **Simplified question or data field** | **Response type / options** |
| 1. Institutional information | Institution identifiers | Institution name | Name of responding School of Public Health or equivalent institution. | Free text |
|  | Institution identifiers | Country | Country where the main campus is located. | Country list / free text |
|  | Institution identifiers | ASPHER membership status | Whether the institution is an ASPHER member. | Pre-coded / yes-no |
|  | Data source | Institutional URL | Official institutional or programme website. | URL |
| 2. Availability of C&H education | Overall availability | Any C&H education | Does the institution offer climate and health education? | Yes / No / Planned / Unknown |
|  | Education level | Vocational or technical training | Is C&H education offered at vocational or technical level? | Yes / No / Planned |
|  | Education level | Bachelor / undergraduate | Is C&H education offered at bachelor or undergraduate level? | Yes / No / Planned |
|  | Education level | Master / postgraduate | Is C&H education offered at master or postgraduate level? | Yes / No / Planned |
|  | Education level | Doctoral level | Is C&H education offered at doctoral level? | Yes / No / Planned |
|  | Education level | Continuing professional development | Is C&H education offered as CPD or short-course training? | Yes / No / Planned |
|  | Future development | Planned C&H offer | Are new C&H education offers planned or under discussion? | Yes / No / Unknown + free text |
| 3. Programme-level information | Programme identity | Programme or course title | Title of each reported C&H course, module, programme, or training activity. | Free text |
|  | Programme identity | Short description | Brief description of the course or programme content. | Free text |
|  | Programme identity | Host programme | If embedded, name of the degree or main programme in which the C&H component is offered. | Free text |
|  | Classification | Education level | Education level of the reported C&H offer. | Vocational / Bachelor / Master / Doctoral / CPD / Other |
|  | Classification | Course type | Whether the offer is standalone or embedded in a broader programme. | Standalone / Embedded / Elective / Mandatory / Other |
|  | Timing and workload | Implementation period or year | When the course or programme was first implemented or offered. | Year / date / free text / unknown |
|  | Timing and workload | Duration | Total duration of the course or programme. | Hours / days / weeks / semester / free text |
|  | Timing and workload | ECTS credits | European Credit Transfer and Accumulation System credits, where applicable. | Numeric / not applicable / unknown |
|  | Qualification | Degree or certificate awarded | Whether completion leads to a degree, certificate, credits, or no formal award. | Free text / categorical |
|  | Delivery | Language of instruction | Language(s) in which the C&H education is delivered. | Free text / multiple choice |
|  | Delivery | Delivery format | Mode of delivery. | In person / Online / Hybrid / Unknown |
|  | Participation | Student enrolment | Approximate number of enrolled or participating students. | Numeric / unknown |
|  | Participation | Enrolment structure | Whether student participation is mandatory or elective. | Mandatory / Elective / Optional / Unknown |
|  | Cost | Course or programme fee | Cost to participate, where applicable. | Numeric in EUR / free text / unknown |
|  | Verification | Programme URL | Link to course, curriculum, syllabus, or programme page. | URL |
| 4. Competency coverage | Governance and policy | Policy frameworks and governance | Does the offer cover climate and health policy, governance, or health-system planning? | Yes / No / Unknown |
|  | Climate-health science | Health impacts and risks | Does the offer cover climate-related health risks and impacts? | Yes / No / Unknown |
|  | Health-system response | Adaptation and resilience | Does the offer cover adaptation, preparedness, or climate-resilient health systems? | Yes / No / Unknown |
|  | Mitigation | Environmental impact and mitigation | Does the offer cover mitigation, low-carbon health systems, or environmental sustainability? | Yes / No / Unknown |
|  | Communication | Communication and stakeholder engagement | Does the offer cover climate-health communication, community engagement, or advocacy? | Yes / No / Unknown |
|  | Ethics and equity | Public health ethics and climate justice | Does the offer address ethics, equity, vulnerability, or climate justice? | Yes / No / Unknown |
| 5. Institutional resources and barriers | Implementation barriers | Limited funding | Is lack of funding reported as a barrier? | Yes / No / Unknown |
|  | Implementation barriers | Limited expertise | Is lack of faculty or expert capacity reported as a barrier? | Yes / No / Unknown |
|  | Implementation barriers | Curricular integration difficulty | Is lack of curriculum space or integration difficulty reported as a barrier? | Yes / No / Unknown |
|  | Implementation barriers | Limited student interest | Is limited student interest reported as a barrier? | Yes / No / Unknown |
|  | Implementation barriers | Limited departmental prioritisation | Is lack of institutional or departmental prioritisation reported as a barrier? | Yes / No / Unknown |
|  | Implementation barriers | Difficulty identifying partners | Is difficulty identifying external partners reported as a barrier? | Yes / No / Unknown |
|  | Implementation barriers | Other barriers | Other reported barriers to implementing C&H education. | Free text |
|  | Resources | Dedicated funding | Is dedicated funding available for C&H education? | Yes / No / Unknown + free text |
|  | Resources | Dedicated experts | Are dedicated institutional experts available for C&H education? | Yes / No / Unknown + free text |

### Supplementary File 1

### Large Language Model (LLM) protocol

The LLM-assisted search was conducted between April and June 2025 using ChatGPT-4o by OpenAI. The model was accessed through a paid ChatGPT subscription with web-browsing/lookup functionality enabled. Default ChatGPT settings were used. No custom-trained model, institutional database, or automated scraping tool was used. Because ChatGPT-4o is a probabilistic model and may change over time, the LLM step was treated as a retrieval-support tool rather than as a reproducible automated search engine.

**Purpose of the LLM step**

The LLM was used to support the web scan by:

1. identifying potentially eligible non-ASPHER and non-responding ASPHER institutions offering public health education;
2. identifying potentially relevant institutional web pages, programme pages, curricula, syllabi, or course descriptions;
3. assisting retrieval in languages other than English by suggesting local-language institutions and links.

The LLM did not make final eligibility decisions. All institutions and programmes were manually verified by the authors.

**Eligibility criteria applied during verification**

Institutions were retained only if they:

1. were located in one of the 53 countries of the WHO European Region;
2. offered degree-awarding public health education at vocational, bachelor, master, doctoral, or continuing professional development level;
3. had verifiable public health programme information available on an official institutional website.

Climate and health education was recorded only when climate-health content was explicitly linked to a public health programme, course, module, syllabus, curriculum, or elective offered by a public health or health-related institution, including frameworks of Planetary and One Health. General institutional sustainability initiatives, research centres, workshops, news items, or informal events were not counted.

**Prompt 1: Country-level institutional identification**

Used separately for each WHO European Region country:

*Please list all institutions that provide public health education at vocational, bachelor, master, doctoral or continuing professional development (CPD) levels based in [COUNTRY]. Create a table with the following columns: Country name, institution name, public health programme name, education level, link for public health programme.*

**Manual verification after Prompt 1**

For each institution returned by the model, the authors manually checked:

1. whether the found institution is an ASPHER member SPH;
2. whether the link led to an official institutional website;
3. whether a public health programme was actually offered;
4. whether the programme was degree-awarding or formally structured;
5. whether the education level matched the classification;
6. whether the institution was a duplicate of an already recorded institution.

Confirmed institutions were marked as “confirmed” in the working spreadsheet. Institutions without verifiable official documentation were excluded.

**Translation procedure**

For websites not available in English, the Google Chrome browser translation function or equivalent browser-based translation was used to translate official institutional pages. Where translated content was unclear, the original-language page was revisited and checked manually. This was particularly relevant for countries with lower availability of English-language institutional documentation, including parts of Eastern Europe, the Caucasus, and Central Asia.

**Prompt 2: Institution-level climate and health search**

Used for each manually confirmed institution or institution list:

*Please list all types of climate and health programmes, courses, modules, or training offered within public health degree/structured education programmes at [LIST OF UNIVERSITIES]. Create a table with the following columns: Institution name, name of climate and health programme/course/training, education level, link to the programme/course/training.*

**Manual verification after Prompt 2**

Each suggested climate and health programme was manually checked against official institutional sources. A programme or course was retained only if:

1. the link was functional or the programme could be found on the official website;
2. the course or programme was linked to public health, health sciences, or a public health degree;
3. the description, syllabus, curriculum, or module title explicitly referred to climate change and health, climate-related health impacts, Planetary Health, One Health, or environmental health with explicit climate content;
4. the activity was structured education, not only a workshop, news item, conference, or informal lecture.

**Prompt 3: Non-responding ASPHER member verification**

For non-responding ASPHER member institutions, an equivalent institution-level prompt was used:

*For [INSTITUTION NAME], identify whether climate and health content is included in publicly available curricula, syllabi, course descriptions, modules, or programme descriptions. Create a table with the following columns: institution name, course or programme name, education level, climate and health content identified, and link to the source.*

**Discrepancy resolution**

When LLM outputs and manual verification differed, manual verification took precedence. Specifically:

- unsupported LLM suggestions were excluded;
- incorrect education levels were corrected;
- duplicate institutions were merged;
- non-degree or informal activities were excluded;
- climate and health classification was only assigned when explicit evidence was found in official programme documentation.

Ambiguous cases were reviewed repeatedly using the official institutional website, translated pages where necessary, and programme-specific documentation. When explicit climate-health content could not be confirmed, the institution was classified as not offering verified climate and health education.

**Assessment of retrieval completeness**

To assess the completeness of the LLM-assisted C&H education retrieval step, the approach was cross-checked against a known ASPHER reference set of 43 Schools of Public Health with known training. The LLM-assisted approach retrieved 42 of 43 institutions, corresponding to a retrieval rate of 98%. This supported the use of the LLM as a screening aid, while final inclusion remained dependent on manual verification.

**Reproducibility note**

Because LLM outputs are probabilistic and model behaviour may change over time, exact replication of the raw ChatGPT-4o outputs cannot be guaranteed. To improve transparency, the model version, access mode, timing, prompt structure, verification rules, discrepancy-resolution process, and manual confirmation criteria are documented here. The final dataset is therefore reproducible through the documented verification protocol and official institutional sources, rather than through reliance on unverified LLM output alone.
